# The common patterns of abundance: the log series and Zipf's law

**DOI:** 10.12688/f1000research.18681.1

**Published:** 2019-03-25

**Authors:** Steven A. Frank

**Affiliations:** 1 Department of Ecology and Evolutionary Biology, University of California, Irvine, CA, 92697-2525, USA

**Keywords:** scaling patterns, ecology, demography, linguistics, probability theory

## Abstract

In a language corpus, the probability that a word occurs *n* times is often proportional to 1/ *n* ^2^. Assigning rank, *s*, to words according to their abundance, log *s* vs log *n* typically has a slope of minus one. That simple Zipf's law pattern also arises in the population sizes of cities, the sizes of corporations, and other patterns of abundance. By contrast, for the abundances of different biological species, the probability of a population of size *n* is typically proportional to 1/ *n*, declining exponentially for larger *n*, the log series pattern.

This article shows that the differing patterns of Zipf's law and the log series arise as the opposing endpoints of a more general theory. The general theory follows from the generic form of all probability patterns as a consequence of conserved average values and the associated invariances of scale.

To understand the common patterns of abundance, the generic form of probability distributions plus the conserved average abundance is sufficient. The general theory includes cases that are between the Zipf and log series endpoints, providing a broad framework for analyzing widely observed abundance patterns.

## Introduction

A few simple patterns recur in nature. Adding up random processes often leads to the bell-shaped normal distribution. Death and other failures typically follow the extreme value distributions.

Those simple patterns recur under widely varying conditions. Something fundamental must set the relations between pattern and underlying process. To understand the common patterns of nature, we must know what fundamentally constrains the forms that we see.

Without that general understanding, we will often reach for unnecessarily detailed and complex models of process to explain what is in fact some structural property that influences the invariant form of observed pattern.

We already understand that the central limit theorem explains the widely observed normal distribution ^1^. Similar limit theorems explain why failure often follows the extreme value pattern ^2, 3^.

The puzzles set by other commonly observed patterns remain unsolved. Each of those puzzles poses a challenge. The solutions will likely broaden our general understanding of what causes pattern. Such insight will help greatly in the big data analyses that play an increasingly important role in modern science.

Zipf’s law is one of the great unsolved puzzles of invariant pattern. The frequency of word usage ^4^, the sizes of cities ^5, 6^, and the sizes of corporations ^7^ have the same shape. On a log-log plot of rank versus abundance, the slope is minus one. For cities, the largest city would have a rank of one, the second largest city a rank of two, and so on. Abundance is population size.

The abundance of species is another great unsolved puzzle of invariant pattern. In an ecological community, the probability that a species has a population size of *n* individuals is proportional to *p ^n^*/ *n*, the log series pattern ^8^. Communities differ only in their average population size, described by the parameter, *p*. Actual data vary, but most often fit closely to the log series ^9^.

In this article, I show that Zipf’s law and the log series arise as the opposing endpoints of a more general theory. That theory provides insight into the particular puzzles of Zipf’s law and species abundances. The analysis also suggests deeper insights that will help to unify understanding of commonly observed patterns.

## Theory

The argument begins with the invariances that define alternative probability patterns ^10, 11^. To analyze the invariances of a probability distribution, note that we can write almost any probability distribution, *q _z_*, as

$$q_{z}=ke^{-\lambda T_{z}},\;(1)$$

in which *T*( *z*) ≡ *T _z_* is a function of the variable *z*. The probability pattern, *q _z_*, is invariant to a constant shift, *T _z_* ↦ *a* + *T _z_*, because we can write the transformed probability pattern in Equation 1 as

$$q_{z}=k_{a}e^{-\lambda (a+T_{z})}=ke^{-\lambda T_{z}},$$

with *k* = *k _a_e* ^– *λa*^. We express *k* in this way because *k* adjusts to satisfy the constraint that the total probability be one. In other words, conserved total probability implies that the probability pattern is shift invariant with respect to *T _z_*.

Now consider the consequences if the average of some value over the distribution *q _z_* is conserved. That constraint causes the probability pattern to be invariant to a multiplicative stretching (or shrinking), *T _z_* ↦ *bT _z_*, because

$$q_{z}=ke^{-\lambda_{b}bT_{z}}=ke^{-\lambda T_{z}},$$

with *λ* = *λ _b_b*. We specify *λ* in this way because *λ* adjusts to satisfy the constraint of conserved average value. Thus, invariant average value implies that the probability pattern is stretch invariant with respect to *T _z_*.

Conserved total probability and conserved average value cause the probability pattern to be invariant to an affine transformation of the *T _z_* scale, *T _z_* ↦ *a* + *bT _z_*, in which “affine” means both shift and stretch.

The affine invariance of probability patterns with respect to *T _z_* induces significant structure on the form of *T _z_* and the associated form of probability patterns. Understanding that structure provides insight into probability patterns and the processes that generate them ^10, 12, 13^.

In particular, Frank & Smith ^12^ showed that the invariance of probability patterns to affine transformation, *T _z_* ↦ *a* + *bT _z_*, implies that *T _z_* satisfies the differential equation

$$\frac{\text{d}T_{z}}{\text{d}w}=\alpha +\beta T_{z},$$

in which *w*( *z*) is a function of the variable *z*. The solution of this differential equation expresses the scaling of probability patterns in the generic form

$$T_{z}=\frac{1}{\beta }(e^{\beta w}-1),\;(2)$$

in which, because of the affine invariance of *T _z_*, I have added and multiplied by constants to obtain a convenient form, with *T _z_* → *w* as *β* → 0. With this expression for *T _z_*, we may write probability patterns generically as

$$q_{z}=ke^{-\lambda (e^{\beta w}-1)/\beta }.\;(3)$$

Turning now to the log series and Zipf’s law, the relation *n* = *e ^r^* between observed pattern, *n*, and process, *r*, plays a central role. Here, *r* represents the total of all proportional processes acting on abundance. A proportional process simply means that the number of individuals or entities affected by the process increases in proportion to the number currently present, *n*.

The sum of all of the proportional processes acting on abundance over some period of time is

$$r=\int_{0}^{\tau }m(t)\text{d}t.$$

Here, *m*( *t*) is a proportional process acting at time *t* to change abundance. The value of *r* = log *n* is the total of the *m* values over the total time, *τ*. For simplicity, I assume *n*_0_ = 1.

Proportional processes are often discussed in terms of population growth ^5, 14^. However, many different processes act individually on the members of a population. If the number of individuals affected increases in proportion to population size, then the process is a proportional process.

Growth and other proportional processes often lead to an approximate power law, *q _n_* ≈ *kn* ^– *ρ*^. However, the exponent of a growth process does not necessarily match the values observed in the log series and Zipf’s law. We need both the power law aspect of proportional process and something further to get the specific forms of those widely observed abundance distributions. That something further arises from conserved quantities and their associated invariances.

The log series and Zipf’s law follow as special cases of the generic probability pattern in Equation 3. To analyze abundance, focus on the process scale by letting the variable of interest be *z* ≡ *r*, with the key scaling simply the process variable itself, *w*( *r*) = *r*. Then Equation 3 becomes

$$q_{r}\text{d}r=ke^{-\lambda (e^{\beta r}-1)/\beta }\text{d}r,\;(4)$$

in which *q _r_*d *r* is the probability of a process value, *r*, in the interval *r* + d *r*. From the relation between abundance and process, *n* = *e ^r^*, we can change from the process scale to the abundance scale by the substitutions *r* ↦ log *n* and d *r* ↦ *n* ^–1^d *n*, yielding the identical probability pattern expressed on the abundance scale

$$q_{n}\text{d}n=kn^{-1}e^{-\lambda (n^{\beta }-1)/\beta }\text{d}n.\;(5)$$

The value of *k* always adjusts to satisfy the constraint of invariant total probability, and the value of *λ* always adjusts to satisfy the constraint of invariant average value.

For *β* = 1, we obtain the log series distribution

$$q_{n}=kn^{-1}e^{-\lambda n},\;(6)$$

replacing *n* – 1 by *n* in the exponential term which, because of affine invariance, describe the same probability pattern. The log series is often written with *e* ^– *λ*^ = *p*, and thus *q _n_* = *kp ^n^*/ *n*. One typically observes discrete values *n* = 1, 2, . . . . The Supplemental material for this article ^15^ shows the relation between discrete and continuous distributions ^16^ and the domain of the variables. The continuous analysis here is sufficient to understand pattern.

For *β* → 0, we have ( *n ^β^* – 1)/ *β* → log *n*, which yields

$$q_{n}=\lambda n^{-(1+\lambda )}\;(7)$$

for *n* ≥ 1. If we constrain average abundance, 〈 *n*〉, with respect to this distribution, then

$$\lambda =\frac{1}{1-1/\langle n\rangle }.$$

For any average abundance that is finite and not small, *λ* → 1, which is Zipf’s law.

Equation 5 provides a general expression for abundance distributions. The log series and Zipf’s law set the endpoints of *β* = 1 and *β* → 0. We can understand the differences between abundance distributions in terms of the parameter *β* by writing the distribution in the generic form of Equation 1, with the defining affine invariant scale

$$T_{n}=\frac{\log n}{\lambda }+\frac{n^{\beta }-1}{\beta }.\;(8)$$

This scale expresses the invariances that define the pattern. At the Zipf’s law endpoint, *β* → 0, the scale becomes 2 log *n* = 2 *r*, when satisfying the constraint that the average abundance, 〈 *n*〉, is sufficiently large.

In this case, with affine invariant scale *T _n_* = 2 *r*, neither addition nor multiplication of process value, *r* ↦ *a* + *br*, alters the pattern. We could have started with this affine invariance, and derived the probability pattern from the invariance properties ^10, 11^.

For the log series endpoint, *β* = 1, the affine invariant scale is

$$T_{n}=\frac{1}{\lambda }\log n+n.$$

The dominant aspect of the scale changes with *n*. For small abundances, the logarithmic scale *r* = log *n* dominates, and for large abundances, the linear scale *n* = *e ^r^* dominates. Many common probability patterns change their scaling with magnitude ^13, 17^.

For log series patterns, the dominance of scale at small magnitude by *r* corresponds to affine invariance with respect to *r*. At larger abundances, the dominance by the effectively linear scale, *n*, corresponds to invariance to a shift in process *r* ↦ *a* + *r*, but not to a multiplication of process, *r* ↦ *br*, because *e ^br^* = *n ^b^* is a power transformation of abundance. Linear scales are not invariant to power transformations. Once again, we could have derived the pattern from the invariances.

In Equation 8, intermediate values of *β* combine aspects of Zipf’s law and the log series. The closer *β* is to one of the endpoints, the more the invariance characteristics of that endpoint dominate pattern.

## Conclusion

This analysis shows how two great and seemingly unconnected puzzles solve very simply in terms of a single continuum between alternative invariances. This approach reveals the simple invariant structure of many common probability patterns.

## Data availability

### Underlying data

All data underlying the results are available as part of the article and no additional source data are required.

### Extended data

Zenodo: Supplemental Material for “The common patterns of abundance: the log series and Zipf’s law”. https://doi.org/10.5281/zenodo.2597895 ^15^.
