# Supplementary material for: The common patterns of abundance: the log series and Zipf's law
Source: F1000Res. 2019 Mar 25;8:334. [Version 1] doi: 10.12688/f1000research.18681.1 (PMC6480937; doi:10.12688/f1000research.18681.1)
Supplement: Peer review history

# Peer review: Reviewer response for version 1

**DOI:** 10.5256/f1000research.20456.r46234

**Published:** 2019-04-29

**Authors:** Neil McRoberts

**Affiliations:** 1 Department of Plant Pathology, University of California, Davis, Davis, CA, USA

This paper continues a series of investigations by Prof Frank into general explanations for the presence of common patterns in the world. Previous work has examined underlying reasons for common probability distributions across a range of types of observation and common size distributions. Here, Steve Frank turns to rank abundance measures.

 There are two central pieces to the analysis presented in the paper. The first derives from E.T. Jaynes' work on the information content of probability distributions and the derivation of probability distributions by considering information constraints on the observed quantity. The second is drawn from Frank's own work (with Eric Smith) on the invariance of the emergent probability distributions to certain scalings of variables, in particular the affine transformation. The same invariance to affine transformation lies at the heart of Frank's previous analyses. 

 For this reviewer, the importance of the paper lies not so much in the technical results (which flow directly as entailments of the algebra given the premises established in equation 1, and the arguments preceding equation 2, but more in the wider issues it raises about observation in general.  In particular, as with the previous papers on related subjects which Frank has written, this paper gives a principled way to derive observed, generic, relationships about rank and abundance that are independent of the physical details of the system being investigated. One possible response to this kind of result is to see it as a consequence of the way that the argument is set up in the premises but I think the way the various parameters are connected with physical properties (albeit it in a generic and hence somewhat abstract way) shows that there is something more fundamental at work here than mathematical slight of hand. Imagine, for example, how strange (to us, here and now) the universe would seem if the informational constraints on probability distributions were not invariant to affine transformation; if, for example, the Poisson distribution described random counts of small numbers of small things, but not small numbers of large things. So, in the same way that understanding what it means for small random counts to follow a Poisson distribution adds to understanding a set of data, it is not a trivial thing (nor a piece of pure phenomenology) for researchers to be able use the general relationship that Frank has derived here, to understand observed rank abundance relationships in terms of the scale at which underlying processes expressing measurable phenomena. 

 In a paper written in such a telegraphic style, Frank does a decent job of connecting the derived relationships with physical examples, but I suspect that anyone who hasn't been following the development of this area of work over the preceding publications, or who isn't familiar with the connections between information and probability, will find the paper concise to the point of abruptness. I hope that Frank plans a synthesis and review of the specific examples that have been published at some later date; although it could be argued that the general synthesis was laid out in earlier papers and the more recent work is unpacking specific case studies.

I have read this submission. I believe that I have an appropriate level of expertise to confirm that it is of an acceptable scientific standard.

# Peer review: Reviewer response for version 1

**DOI:** 10.5256/f1000research.20456.r46235

**Published:** 2019-04-17

**Authors:** Scott E. Page

**Affiliations:** 1 Department of Political Science, Center for the Study of Complex Systems, University of Michigan, Ann Arbor, MI, USA

I found this article to be fascinating and elucidating but also a bit frustrating to read. The central claim of the article is that one can construct a family of distributions such that Zipf's law and species abundance are the endpoints of a more general process.

 For that result to be interesting, the result has to be **for a general process** and not **for a family of distributions.**

 The latter is easy. I just say, "here is a family of distributions, f(x) = x^{-a}” and then say that at one endpoint a=1 I have a species area law and at the other endpoint a=2, I get Zipf's law.  

 The contribution of the article lies in convincing us that the paper has done something other than an elaborate change of variables that simply restates that result through obfuscation.

 So what does the paper do? The paper shows that if we restrict attention to probability patterns (by the way, it would be nice if "pattern" were formally defined) that are invariant to affine transformations then we have a specific form given by equation (3).

 Given the form in equation (3), the author then claims that n represents pattern and r represents process. This needs to be elucidated. 

 For the main result, once we have invariance to affine transformation we get the differential equation with

 dT_z/dw  = \alpha + \beta T_z

 From here, why doesn't it just follow that if \beta = 0, we have something that's going to fall off with a common invariant scale and for \beta = 1 the invariant scale changes with n.

 The conclusion of the paper needs to be expanded. As a reader, I need a richer explanation for how the approach "reveals the simple invariant structure" of common probability distributions. In the conclusion, we should be given more intuition for how the holding the average abundance constant drives the results. Also, it would be nice to have more insight into what would cause a system to have more or fewer proportional processes acting on it.

 Quibble:

 Why isn't r a function of tau—the period of time?

I have read this submission. I believe that I have an appropriate level of expertise to confirm that it is of an acceptable scientific standard.

# Response

**Published:** 2019-04-22

**Authors:** Steven Frank

**Affiliations:** Department of Ecology & Evolutionary Biology, University of California, Irvine, USA

I appreciate the thoughtful comments from Scott Page. The review and my reply are part of the final published version. So, I will use this space to develop my comments, leaving the original manuscript unchanged. I quote the first few words of each reviewer comment in bold, and then follow with my reply.

**For that result to be interesting, the result has to be for a general process and not for a family of distributions.**

I partially agree. It is very interesting to understand how a general process relates to a family of distributions. I am currently pursuing that by studying the species abundance problem in ecology in more detail as an example. In my new work, I show how various well known general processes, such as neutral theory, relate to an abstract family of distributions characterized by simple invariances. My new work will show a much simpler way in which to understand the relations between process and pattern in ecology than is currently the case in the literature of that subject. I think that new work will help a bit with regard to this question, because the log series and Zipf’s law are special cases of a broader family of distributions that also includes the lognormal. The current article is a step on the way to the more ambitious study.

However, I also partially disagree. Identifying the invariant structure of probability patterns is by itself useful. It guides one in more focused projects and provides a way to understand what is expected and what is not. Further, one can specify a general process that leads to a Gaussian distribution, but that process will not be a full understanding of the Gaussian distribution, just one instance of a process that associates with that pattern. There are other general processes that are distinct but also converge to the Gaussian. So, we need to understand both the different types of general process and the abstract aspects of the Gaussian that unify all conforming general processes.

The current article is entirely on the abstract side. That is a necessary but not sufficient component. I agree that more needs to be done and am working on that in the ecology application mentioned above. I hope to contribute along those lines in my future work.

**So what does the paper do? The paper shows that if we restrict attention to probability patterns (by the way, it would be nice if "pattern" were formally defined) ...**

No widely agreed definition of “pattern” exists, which is interesting. I believe that “pattern” and “invariance” are the same thing, but that remains an open issue.

**Given the form in equation (3), the author then claims that n represents pattern and ...**

My use of n as pattern simply describes what people have typically measured and reported as a pattern. In other words, people have measured population sizes and reported those data as a pattern. My claim that r corresponds to process reflects the general agreement that populations change by birth, death, migration, and other processes that act multiplicatively, again a description of the common understanding. For example, the neutral theory in ecology, which has become a dominant approach to the study of ecological process, is about demographic processes that act proportionally or multiplicatively. So, both by intuition and by consensus, I have adopted r as associated with process.

**From here, why doesn't it just follow that if \beta = 0, we have something that's going to fall off with a common invariant scale and for \beta = 1 the invariant scale changes with n.**

I do not understand the question. The point of the differential equation is that we can find a new scale, w, that is shift invariant but not stretch invariant with respect to probability pattern. As \beta goes to zero, w becomes both shift and stretch invariant (affine invariant). It turns out that \beta is a sort of curvature that describes departure from stretch (multiplicative) invariance. As an observation, working with w has turned out to provide a key way in which to unify diverse probability distributions within a single common system, suggesting an invariant structure that unifies commonly observed probability patterns. That was the topic of several of my prior publications. Here, I was just using some of the prior insight to try and understand the nature of the log series and Zipf’s law and perhaps something about how those distributions arise. I will expand on that in a future manuscript, see next comment.

**The conclusion of the paper needs to be expanded. As a reader, I need a richer explanation ...**

First, my prior publications discuss invariant structure and its consequences in an abstract way. But I think that is not the issue that is being asked about here. So, second, I am currently finishing a new manuscript that extends this current article in several ways. My new manuscript focuses on invariance in ecological pattern. By emphasizing a particular application and its associated literature, I am able more explicitly to get at some of the issues that are too vague in the current manuscript. For example, I will consider directly the role of average values by relating maximum entropy and invariance approaches explicitly in the context of ecological applications. I think this will help some. Many of the other comments raised by the reviewers also come up in the new work, suggesting that there are some obvious missing steps here that need further attention. These issues cannot be resolved in a few paragraphs, so I am going to wait until I finish the new work before trying to address these problems. I apologize for putting off thoughtful and important comments, but I need more time to complete the new work before I can give good answers.

**Why isn't r a function of tau—the period of time?**

It is. Whether that matters depends on the particular question. One aspect is that, as long as tau is taken as a fixed value, such as generation time in a discrete generation model of populations, then one can take r directly without concern about varying tau. Alternatively, if one has reason to consider tau as varying, then it may matter for certain aspects. I have not looked into that. I agree that it would be worthwhile to understand this issue more clearly.

# Peer review: Reviewer response for version 1

**DOI:** 10.5256/f1000research.20456.r46236

**Published:** 2019-04-17

**Authors:** Jose Lobo

**Affiliations:** 1 School of Sustainability, Arizona State University, Tempe, AZ, USA

1. The manuscript addresses the relationship between two probability distributions that, although originated in specific research domains, have gone on to be widely used as representations of growth processes.
2. The mathematical derivations are clear.
3. The conclusion that "two great and seemingly unconnected puzzles solve very simply in terms of a single continuum between alternative invariances. This approach reveals the simple invariant structure of many common probability patterns." clearly follows from the exposition and is a useful contribution.
4. The usefulness and scope of the conclusion would be strengthened if the author considered another distribution which arises often in the explorations of growth processes: the log normal.
5. It would also strengthen the usefulness of the manuscript if the author were to expand on "this approach reveals the simple invariant structure of many common probability patterns.", in particular recapitulating what is the invariant structure.
6. Having connected two widely used distributions, what sort of research questions can now be addressed? How can the invariant structure linking two distributions be used in contexts other than Zipf's law or species distributions?

I have read this submission. I believe that I have an appropriate level of expertise to confirm that it is of an acceptable scientific standard.

# Response

**Published:** 2019-04-22

**Authors:** Steven Frank

**Affiliations:** Department of Ecology & Evolutionary Biology, University of California, Irvine, USA

I appreciate the thoughtful comments from Jose Lobo. The review and my reply are part of the final published version. So I will use this space to develop my comments, leaving the original manuscript unchanged. I quote the first few words of each reviewer comment in bold, and then follow with my reply.

**The usefulness and scope of the conclusion would be strengthened if the author considered another distribution which arises often in the explorations of growth processes: the log normal.**

Copying my reply to Luis Bettencourt’s comment on the same topic: ****I have a new unpublished manuscript that unifies the log series, Zipf’s law, and the lognormal. A single additional invariance leads to a unified distribution that includes all of those distributions as special cases and also some intermediate forms that commonly arise in certain empirical examples. Thus, I agree with the importance of this comment, but withhold further details until I can complete my new work.

**It would also strengthen the usefulness of the manuscript if the author were to expand on "this approach ..."**

I agree that the current manuscript is rather terse about this issue. However, I have written several prior manuscripts that extensively develop this aspect, cited in the current publication. I prefer to keep the current manuscript short and focused on the new aspect related to the log series and Zipf’s law, and refer to earlier publications for the background.

**Having connected two widely used distributions, what sort of research questions ...**

I think the key advance will not come until more can be said about linking dynamic models to the abstract invariant structures. Copying my reply to Luis Bettencourt’s review: I agree that connecting dynamic models of process to the invariances that set pattern is the great missing piece in this work. Ultimately, an invariance perspective can only achieve its full usefulness if one can compare and empirically test alternative hypotheses about mechanistic processes. That might, for example, require one to identify the particular aspect within a mechanistic set of processes that ultimately defines the invariance that dominates pattern. Then, by comparing different mechanistic models, one can reduce that comparison to the contrast between alternative component processes that dominate invariance, and so develop a more focused empirical test. I am not yet certain how to make these connections between abstract invariances and mechanistic dynamical models in the most meaningful way, so I have refrained from writing about these issues. This is a primary topic for future work.

# Peer review: Reviewer response for version 1

**DOI:** 10.5256/f1000research.20456.r46232

**Published:** 2019-04-15

**Authors:** Luís M. A. Bettencourt

**Affiliations:** 1 Department of Ecology and Evolution, University of Chicago, Chicago, IL, USA; 2 Santa Fe Institute, Santa Fe, NM, USA

This manuscript approaches the origins of two particularly important distributions describing abundances in biological and social populations from the perspective of mathematical invariances of their mathematical forms. 

 The author shows in this way that Fisher’s log series distribution and Zipf’s “law” can arise in different limits of the same parameter, characterizing a family of affine transformations that includes translations and scale transformations of growth rates.

 The mathematical derivation is clear and elegant, so that the manuscript makes an important contribution to formal models deriving these abundance distributions. 

 What I think would improve the manuscript is greater contact with other methods for the derivation of these same distributions of abundance and an expanded discussion of limits. 

 Specifically: 

  

1. The relationship between population dynamics and invariances of the abundance (or rate) distributions could be made a little more explicit: Population dynamics models (in analogy to other dynamical systems) are mappings, tracing explicit variable transformation over time, such as changes of “position” (translations, r-> a + r), or dilations (r -> b r). Asking for invariances of distributions under these dynamical transformations is the usual way to derive the distributions as steady state abundances. Power laws, such as Zipf’s law, are invariant under (stochastic) dilations, for example, while Fisher’s log series are invariant under other simple types of population dynamics (as in Volkov et at ^1^). I’d appreciate a bit more discussion bridging these two approaches.   
2. As, the author shows the derivation of Zipf’s law requires not only a parameter choice (beta -> 0) but also the limit of the average abundance -> infinity. Without the latter, the power law exponent won’t be Zipf’s. In dynamical derivations of Zipf’s law one asks instead that geometric random motion of the population abundances, is subjected to a (“reflecting”) boundary condition for small population sizes that stops them from getting too small, as in [5]. Under what circumstances are these two additional requirements (besides transformational invariances under multiplicative growth) equivalent? They seem to have a different character as one is a limit, while the other a boundary condition—is the limiting condition on the average the most general condition?
3. It would be interesting to describe the conditions (in terms of beta and any limits or time dependence on averages) for deriving the third distribution often invoked to describe the same abundances, the log-normal, in terms of the reasoning about invariances advanced here. This is discussed to some extent in previous work by the author, in reference [12]. I think its inclusion and discussion would benefit the current manuscript.

I have read this submission. I believe that I have an appropriate level of expertise to confirm that it is of an acceptable scientific standard.

## References

[1] VolkovIBanavarJRHubbellSPMaritanA: Patterns of relative species abundance in rainforests and coral reefs. *Nature*.2007;450(7166) : 10.1038/nature06197 45-9 10.1038/nature06197 17972874

# Response

**Published:** 2019-04-22

**Authors:** Steven Frank

**Affiliations:** Department of Ecology & Evolutionary Biology, University of California, Irvine, USA

I appreciate the thoughtful comments from Luis Bettencourt. The review and my reply are part of the final published version. So I will use this space to develop my comments, leaving the original manuscript unchanged. I quote the first few words of each reviewer comment in bold, and then follow with my reply.

**The relationship between population dynamics and invariances of the abundance (or rate) distributions could be made a little more explicit: ...**

I agree that connecting dynamic models of process to the invariances that set pattern is the great missing piece in this work. Ultimately, an invariance perspective can only achieve its full usefulness if one can compare and empirically test alternative hypotheses about mechanistic processes. That might, for example, require one to identify the particular aspect within a mechanistic set of processes that ultimately defines the invariance that dominates pattern. Then, by comparing different mechanistic models, one can reduce that comparison to the contrast between alternative component processes that dominate invariance, and so develop a more focused empirical test. I am not yet certain how to make these connections between abstract invariances and mechanistic dynamical models in the most meaningful way, so I have refrained from writing about these issues. This is a primary topic for future work.

**As, the author shows the derivation of Zipf’s law requires not only a parameter choice ...**

First, one just needs average abundance to be not small to get an exponent that is essentially equivalent to Zipf’s law and sufficient for empirical comparison. Second, I agree that it would be useful to consider the relations between boundary conditions in dynamics and simplified invariance models. I don’t know the answer. It would be a useful topic for future work.

**It would be interesting to describe the conditions (in terms of beta and any limits or time dependence on averages) for deriving ...**

I have a new unpublished manuscript that unifies the log series, Zipf’s law, and the lognormal. A single additional invariance leads to a unified distribution that includes all of those distributions as special cases and also some intermediate forms that commonly arise in certain empirical examples. Thus, I agree with the importance of this comment, but withhold further details until I can complete my new work.
